# Emotional intelligence assessment in a graduate entry medical school curriculum

**DOI:** 10.1186/1472-6920-13-38

**Published:** 2013-03-07

**Authors:** Eva M Doherty, Patricia A Cronin, Gozie Offiah

**Affiliations:** 1 National Surgical Training Centre & Graduate Medical School, Royal College of Surgeons in Ireland, 121, St Stephens Green, Dublin 2, Ireland; 2 Graduate Medical School, Royal College of Surgeons in Ireland, Ballymoss Road, Sandyford Industrial Estate, Dublin 18, Ireland

**Keywords:** Emotional intelligence (EI), Empathy, Gender, Curriculum

## Abstract

**Background:**

The management of emotions in the workplace is a skill related to the ability to demonstrate empathic behaviour towards patients; to manage emotional reactions in oneself and to lead others as part of a team. This ability has been defined as emotional intelligence (EI) and doctor’s EI may be related to communication skills and to patient satisfaction levels. This study reports on the use of two assessments of EI as part of a course on Personal and Professional Development (PPD) in a graduate medical school curriculum.

**Methods:**

Fifty one graduate entry medical students completed an eight session course on PPD between December 2005 and January 2006. Students completed two measures of EI: self-report (EQ-i) and ability (MSCEIT V2.0) over a two year study period. The data gathered were used to explore the relationship between self-report and ability EI and between EI and student demographics, academic performance and change over time.

**Results:**

Analysis of the EI data demonstrated that self-report EI did not change over time and was not related to ability EI. Females scored higher than males on a number of self-report and ability EI scores. Self-reported self-awareness was found to deteriorate in males and females over time. High self-reported EI was found to be associated with poor performance on clinical competency assessments but with good performance on a number of bio-medical knowledge based assessments.

**Conclusions:**

This report concludes that assessments of EI can be incorporated into a medical school curriculum as part of a PPD programme and that the concept of EI may be associated with performance in medical school.

## Background

Emotional intelligence (EI) is recognised to be an important component of the doctor–patient relationship and has been demonstrated to be related to the level of trust and satisfaction felt by the patient towards the doctor [1-3]. The ability to demonstrate empathy is recognised to be an important emotional skill and studies of empathy in the medical student/trainee indicate that empathy deteriorates over the course of training and work [4-7]. Personality, stress, sleep deprivation, depression and burnout are just some of the possible mediating factors [8-11]. Medical educators have a responsibility to provide training for students to develop the ability to better manage their emotional responses to stressors and to prevent the attrition of empathy skills. A recent systematic review sought to investigate whether such training programmes existed and if they were effective. The authors identified 26 studies which focussed on empathy skills training and demonstrated positive outcomes [12]. Similarly, several studies report the effectiveness of stress-reduction programmes for medical students [13-15]. Thus the evidence indicates that interventions can be successful in reducing the effects of stress and in enhancing medical students’/ junior doctors’ empathy skills.

The concept of EI has been incorporated into certain interpersonal skills sections of the Australian medical aptitudes test [16] and the evidence regarding EI and internationally recognised medical competencies has been recently systematically reviewed [17]. EI has been identified as one of several important concepts that could help move the culture of medical education ahead by creating a better learning, working and caring environment [18]. The evidence that EI may be a factor in medical competency is indicated by a recent study which found that self-rated emotional functioning and ability scores were found to be associated with scores on assessments of communication and physical examination skills in a sample of graduate medical students, [19] and studies of non-medical third level students have proposed that EI may be related to academic ability [20-22]. EI has been shown to be higher in female medical students at entry to medical school [23,24] but to deteriorate over the course of medical training [25]. While a number of studies report on the use of EI in medical schools, only one study could be found which attempted to incorporate EI self-assessment and training into the medical curriculum [26]. The study sub-contracted an external agency to run the training course and although an increase in EI scores was demonstrated following attendance, only 34% of the cohort attended the complete course. Another study conducted at Peninsula graduate medical school UK, reported that the majority of students welcomed the opportunity to learn about EI and their self-rated emotional competencies [27] however very few studies to date have reported on how to design training programmes to provide students with the opportunity to learn explicitly about their own EI and such training programmes are well established in the business world [28,29].

Finally, the majority of studies of medical students to date have used self-report measures of EI however recently it has been shown that it is possible to use an ability measure of EI in a medical school [30] although it is likely that self-report and ability measures may be measures of different aspects of EI [31].

### What is EI?

EI has been defined both as an ability and a trait and as a mixture of both. The ability definition describes EI as the ability to monitor one’s own and others’ feelings and emotions, to discriminate among them and to use this information to guide one’s thinking and actions [32]. Trait theorists define EI as a constellation of emotion-related self-perceptions and dispositions, assessed through self-report [33]. Mixed model theories define EI as an array of non-cognitive capabilities, competencies and skills [34].

### Measurement of EI

The trait models and mixed-models advocate the use of self-report as their mechanism of measurement, inferring that individuals who state that they function at various levels actually do. Examples of these models include the Emotional Quotient Inventory (EQ-i: [34]), the Emotional Competence Inventory (ECI: [35]), the Emotional Intelligence Scale (EIS: [36]) and finally the Trait Emotional Intelligence Questionnaire (TeiQue: [33]).

The Mayer-Salovey-Caruso Emotional Intelligence Test (MSCEIT V2.0: [37]) is an ability measure and assesses the four branches of Mayer and Salovey’s EI ability model [38,39]. The MSCEIT V2.0 yields a profile of scores describing an individual’s ability to perceive, use, understand and manage emotions. The topic of EI in psychology is a relatively new and controversial one and one of the difficulties is that self-report measures of EI do not correlate with ability measures of EI [40,41]. Notwithstanding the uncertainty in the EI literature, the concept of EI nevertheless offers the medical educator an attractive tool for the facilitation and development of the so called “non-cognitive” abilities of the medical student/trainee [42,43].

This study investigated the following research questions in a sample of graduate medical students:

Does EI change over the course of medical training?

Is there a gender difference in EI?

Are self-report EI scores associated with ability EI scores?

Is there a relationship between EI scores and academic achievement?

## Methods

The first EI assessment (T1) was administered as part of a PPD course which took place between October and December 2006. The second EI assessment (T2) took place between the months of January and May 2008 when the students were in their second year of graduate medical programme. In the first year of the graduate medical programme, students’ complete 12 modules encompassing systems based approach to medicine. The PPD course was given as part of one of these modules – Health Behaviour and Society module. Permission to conduct the study and to analyse student EI data was granted by the Research Ethics Committee at the Royal College of Surgeons in Ireland.

Fifty one graduate entry medical students attended an eight week course on PPD. Each week, teaching took place during one three hour session. Student assessment was by means of two written assignments which counted for 10% of the final mark in the Health Behaviour and Society module, one of six modules in the first semester of the first year of the medical curriculum. In addition, students maintained an eportfolio worth 20% of the final mark in the Clinical Competencies module. Students also completed other PPD related activities as part of the standard curriculum in their first year such as team projects, integrated clinical communication skills practice and clinical placements. The topics in the eight week PPD course were as follows:

1. Professionalism and the eportfolio (two sessions).

2. Stress and stress management (three sessions).

3. Leadership and emotional intelligence (three sessions).

Teaching methods incorporated didactic presentations, small and large group activities and discussion. (See Additional file 1 for an outline of content and teaching methods). Self-report EI was assessed as part of the PPD course at T1. Two years later (T2), students were requested to complete the self-report EI once more and an ability EI measure. Only the EI component of the programme and the results of the analysis of the EI data will be described.

### EI components of the PPD programme

EI was presented to the class as an important component of communication and leadership abilities and self-care. The first session, Leadership one, consisted of a slide presentation and discussion. Students subsequently received instructions on how to complete the EQ-i self-report measure online and assured of confidentiality. Students’ permission to publish annonymised results was requested in writing. Only the principal author (ED) had access to EI profiles in addition to the students.

Prior to the second session (Leadership two) each student completed the online EI assessment and received an individualised resource report from the principal lecturer; a practising Clinical Psychologist (ED). Group feedback was provided during the Leadership two session and students were invited to contact the lecturer for individual feedback if desired. The third session on leadership was designed to demonstrate how the EI profiles were applicable to various aspects of their professional roles in the future as practitioners and leaders with tips for developing EI competencies.

### Student assessment

Assignment two of the PPD module required the student to study their individual EI profiles and conduct a SWOT (Strengths, Weaknesses, Opportunities and Threats) analysis. The aim of the assignment was to facilitate students’ reflection on their EI profile and on strategies to enhance emotional awareness and understanding.

### Description of EI measures used in PPD course

#### Emotional Quotient Inventory (EQ-i)

Two measures of EI were used. The first was the EQ-i [34]; a self–report measure completed online through a website (http://www.mhsassessments.com) although a pen and paper version is available. The measure comprises of either 133 or 125 brief items, employing a five-point response set (ranging from “Not true of me” to “True of me”) and takes approximately 30–40 minutes to complete with no imposed time limits. The EQ-i renders four validity scale scores, a total EQ score, five composite scale scores and 15 EQ subscale scores. EQ-i raw scores are converted into standard scores based on a mean of 100 and a standard deviation of 15 (similar to IQ scores). This score conversion allows for comparison with the normative group and also with other EI measures using standard scores such as the second measure used in this study.

Internal consistency scores for the EQ-i (Cronbachs alpha) range from r = .69 (Social Responsibility) to r = .86 (Self-Regard) with an overall average coefficient of r = .76 for the full scale score. Second-order confirmatory factorial analysis provides support for the five composite scales described in the test manual. Correlations coefficients ranged from r = .30 to r = .70.

**Figure 1:**
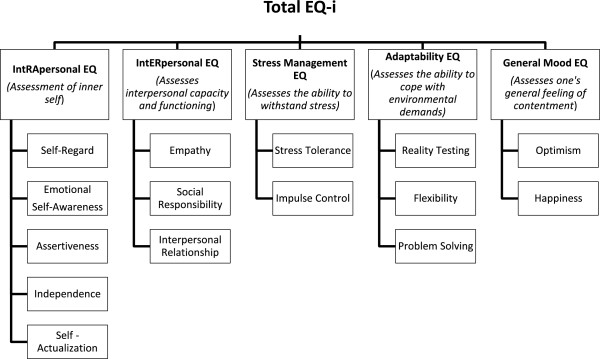
Subscales of the EQ-i and a brief description.

The authors conclude that these results support the tenet that the EQ-i subscales are measuring the constructs that they were intended to measure but are not so high as to suggest that the EQ-i is a duplication of existing inventories. Other forms of validity are presented in detail in the manual. A revised edition of the EQ-i has been made available by the agency since June 2011 and a 360° administration is also available. Figure 1 shows the subscales of the EQ-i and a brief description.

#### Mayer-Salovey-Caruso emotional intelligence test (MSCEIT V2.0)

**Figure 2:**
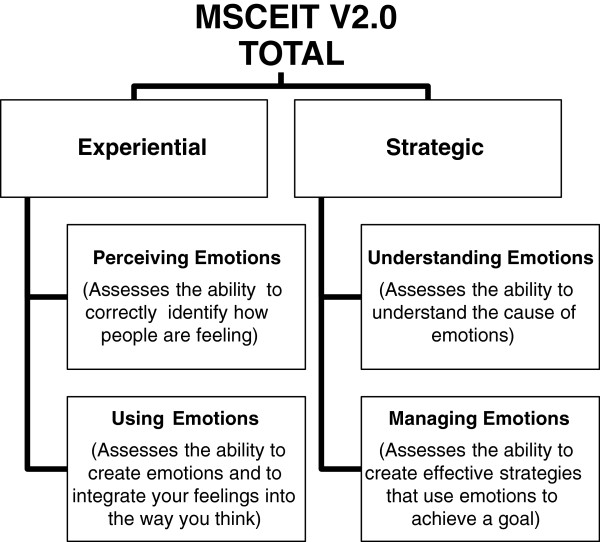
Subscales of the MSCEIT V2.0 and a brief description.

The MSCEIT (pronounced “Mes-keet”), is an ability-based instrument which measures how well people perform emotional tasks and solve problems involving emotional situations [37]. In some subscales, the test presents a series of photos and pictures and the respondent is required to identify the emotions portrayed. Other subscales ask the respondent to choose the most appropriate strategy to solve an interpersonal situation. The measure is distributed by the same agency in the same way as the EQ-i described previously (http://www.mhsassessments.com). There is a paper and pen version available however online administration is the usual method. The measure comprises of 141 test items and takes between 30–45 minutes to complete. The main scores that the MSCEIT V2.0 produces are an overall total EIQ score, two area EIQ scores, four branch EIQ scores and eight task scores. There are also three supplemental scores: a scatter score, a positive–negative bias score and an omission rate. Reliability coefficients for the full scale score are quoted in the manual and demonstrate good internal consistency for the full scale score (r = .91) with subscale scores ranging from r = .74 to r = .89. Good evidence for face, content, factorial and predictive validity is presented in the manual. Figure 2 gives the subscales of the MSCEIT with a brief description.

## Results and discussion

### Statistical analysis

Data were analysed using SPSS version 18.0. As the data were normally distributed, independent sample t-tests were conducted to compare the means of EQ-i and MSCEIT V2.0 scales with regards to gender and age. The comparison of means between the students’ scores at first assessment and at follow up was conducted using the paired t-test. Pearson’s product–moment correlation was used to explore the relationship between continuous variables.

### Respondents

Forty nine (98%) of the 51 students in the class completed the EQ-i measure at T1 and gave consent for their anonymised data to be analysed and results published. At T2, thirty three (67%) students completed the EQ-i and the MSCEIT V2.0. The mean EQ-i at T1 was 105.6 (range 73 – 131). Differences between mean EQ-i scores and proportion of males/females, ages, were investigated using t-tests and chi-square analysis and none were found (Table 1). However, Pearsons correlation coefficient indicated that student age was significantly positively associated with EQ-i scores (r = .36, p ≤ .05) but not with MSCEIT V2.0 scores. There was no difference found between the EQ-i scores of those students at T1 who did not complete the second EI assessment and the EQ-i scores of the students who did.

**Table 1:** Sample characteristics at first (T1) and second (T2) assessment: sex, mean ages (standard deviation), mean EI (standard deviation)

**T1**				**T2**				
**n = 49**				**n = 33**				
**Sex**	**N**	**Age**	**EQ-i**	**Sex**	**N**	**Age**	**EQ-i**	**MSCEITV2.0**
**Female**	31	24.03(1.65)	105.87(10.17)	**Female**	18	26.28(1.67)	109.33(11.22)	107.47(13.12)
**Male**	18	23.82(2.01)	104.35(9.76)	**Male**	15	25.71(1.77)	104.33(12.75)	101.04(15.51)

Note: All means non significantly different.

The following questions were investigated:

#### Does EI change over the course of medical training?

There was no significant difference in self-report total EQ-i scores from T1 to T2. In subscale analysis however, there was a significant decrease in EQ-i Self-Awareness scores: (M = 107.7, SD = 15.9) versus (M = 103.8, SD = 13.9, p ≤ *.*05). The mean difference in the EQ-i Self-Awareness scores was 3.91 points with a 95% confidence interval ranging from 0.254 to 7.564. The eta-squared statistic (.13) indicated a moderate to large effect size.

Scores on one of the self-report EQ-i subscales (Reality Testing) for males increased significantly from T1 to T2. (M = 98.9, SD = 8.9) versus (M = 106.8, SD = 13.7, p ≤ *.*05); while scores on the interpersonal skills section, a section of the EQ-i comprising of three subscales (Empathy, Social Responsibility and Interpersonal Relationship) deteriorated between T1 and T2 in females (M = 113.2, SD = 10.7) versus (M = 104.6, SD = 10.2, p ≤ .01).

#### Is there a gender difference in EI?

**Figure 3:**
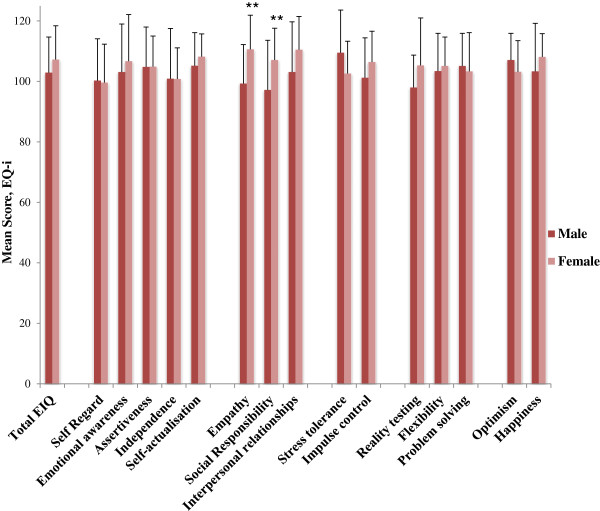
Comparison of self-reported emotional intelligence scores (EQ-i) according to gender at the first assessment (T1).

While no significant difference was found on total EQ-i scores between male and female students, differences were found in favour of females on some of the subscale scores. Comparison of means indicated that female students scored significantly higher than males on self-reported empathy (M = 110.6, SD = 11.4) versus (M = 99.3, SD = 12.9, p ≤ .01) and social responsibility (M = 107.1, SD = 10.4) versus (M = 97.2, SD = 16.4, p ≤. 01) (Figure 3).

In the analysis of EQ-i scores at T2, no further differences in male versus female self-reported total EQ-i scores were found. Female students maintained their significantly higher empathy and social responsibility scores at the second time point.

**Figure 4:**
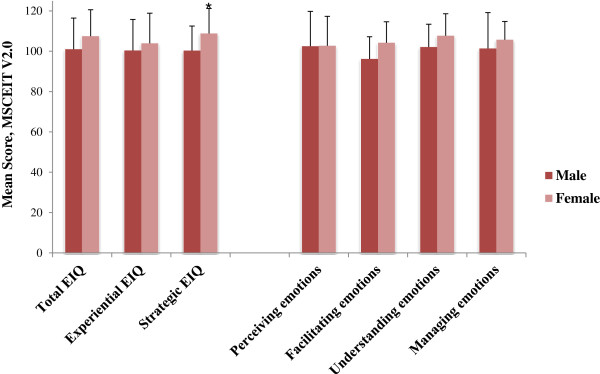
Comparison of ability emotional intelligence scores (MSCEIT V2.0) according to gender at the second assessment (T2).

With regard to the ability measure of EI, female students scored significantly higher than the male students on the Strategic EIQ area, (M = 108.8, SD = 10.4) versus (M = 100.3, SD = 10.9, p ≤ *.*05) (Figure 4).

#### Are self-report EI scores associated with ability EI scores?

To investigate if there was a statistically significant association between self-reported EI scores (as measured by the EQ-i) and ability EI scores (as measured by the MSCEIT V2.0), a Pearson-product moment correlation was computed and was not significant.

#### Is there a relationship between EI scores and academic achievement?

Scores on all module assessments were compared to EQ-i scores at T1 using Pearson’s correlation coefficient. EQ-i scores were negatively associated with performance on the Clinical Competency (CC) modules (r = −.38, p ≤ .01) such that students who rated themselves as possessing good emotional competencies were more likely to obtain low scores on these assessments. These modules are assessed by means of OSCE (Objective Structured Clinical Examinations), designed to assess both clinical and communication skills. Conversely, scores on another EQ-i subscale (Impulse Control) were found to be positively associated with scores on four other module assessments; Health Behaviour and Society, Evidence Based Health, Haemopoietic and Immune Systems, Genitourinary and Endocrine (all r’s between .28 and .50).

Correlation coefficient scores for EQ-i, MSCEIT V2.0 and academic performance were compared at T2. Once again an association between high EQ-i scores and poor performance on the CC module were found with medium negative correlations demonstrated between two of the EQ-i subscale scores and OSCE scores (Inter-personal Relationship: r = −.39, p ≤ .05; Self-Regard: r = −.39, p ≤ .05). Similarly, medium negative correlations were also found between two EQ-i subscales (Interpersonal relationship: r = −.39, p ≤ .05; Happiness: r = −.35, p ≤ .05) and performance on one of the other modules, the Biology and Epidemiology Disease module. These results suggest that students who rate themselves as happier with good interpersonal relationships and high self-regard were more likely to obtain lower marks on these assessments.

## Conclusions

The purpose of this study was to demonstrate that the concept of EI can be included in a PPD course within the context of a systems based medical curriculum. This represents a first step for this graduate medical school in addressing the difficult topic of professionalism and the mechanisms, which can be used to teach and assess it. The EI data gathered within the context of the course activities were statistically analysed and a number of significant associations between EI scores and gender, time and academic assessments were identified.

The finding that both male and female students rated their self-awareness competency at a lower level at T2 compared to T1 is in agreement with the evidence discussed above that empathy skills deteriorates over the course of medical training. Female students demonstrated superior emotional competencies on some of the self-report and ability EI scores. However females were also more likely to report deterioration in some of these emotional competencies over time. It may be that the medical education environment encourages females to adapt their interpersonal skills and emotional competencies to become more similar to their male counterparts.

A number of self-report EI competencies were associated negatively with performance on some of the academic modules and this warrants further investigation. There is evidence that self-rated EI is closely associated with personality [33,41] and so this may be a reflection of the nature of the relationship between personality and academic performance in medical training [44]. The finding that those students who rated themselves highly on certain emotional competencies were also more likely to obtain low scores on assessments of their clinical and communication skills may be an indication of their lack of insight into their true abilities or may be to do with the validity of the assessments themselves. An inverse relationship between certain self-report EI competencies and performance on clinical OSCEs was identified again at T2 and this supports the possible association between lack of insight and poor clinical performance. In contrast, strong to medium positive correlations between the EQ-i subscale Impulse Control and four module scores were demonstrated at T1 such that a perceived high ability to manage frustration was associated with high scores on these knowledge based assessments. However at T2, an inverse association between certain self-report EI competencies and performance on one of the knowledge based modules was found which indicates that the relationship between self-reported EI and actual academic performance is not straightforward and should be investigated further.

Ability EI and self-report EI did not correlate and there were no associations between the ability EI scores and academic performance indicating once more that these two conceptualisations of EI are not assessing the same aspect of emotional competency. The sample size at T2 which compared the ability EI scores with academic performance was small (n = 33) and so the analysis was vulnerable to a Type II error (i.e. failure to find an association when one exists).

There are a number of limitations to this study. The analysis was conducted within the practical limitations necessarily encountered within the confines of the demands of a medical curriculum. The study thus took advantage of the availability of the EI data to explore pertinent questions concerning EI and medical students against performance. The findings are very preliminary and will require replication in a more methodologically robust study. The size of the samples at both assessment points were small and in particular at T2. Finally, a bonferroni correction was not applied and so it is possible that some of the significant correlations occurred by chance. Replication with more participants is required to investigate the relationships identified.

Though a very small cohort, our study has shown that there are significant changes in several subscales of the self-reported and ability scores of EI and the subsequent correlations with academic performance. We have also shown that it is possible to include student EI assessment in a PPD programme within a medical curriculum. We thus invite other medical educators to adapt this PPD programme including EI assessment as outlined to suit their own needs and to evaluate it within the context of their medical curricula.

## Competing interests

The authors declare that they have no competing interests.

## Authors’ contributions

ED carried out the design of the study and acquisition of data. ED also collated the analysis and interpretation of the data and the drafting of the manuscript. PC participated in the analysis of data and in drafting the manuscript. GO was involved in revising the manuscript critically. GO was also involved in preparing the tables and figures as well as in preparing and analysing some of the data. All authors read and approved the final manuscript.

## Authors’ information

ED is Director of Human Factors and Patient Safety at the National Surgical Training Centre at the Royal College of Surgeons in Ireland (RCSI). Prior to this appointment in 2009, Eva was Senior Lecturer/Clinical Psychologist at the Graduate School of Medicine at RCSI.

PC was a clinical tutor in 2009 on the graduate entry medical programme with the Royal College of Surgeons in Ireland. She is also a specialist registrar in general surgery.

GO is a surgical trainee and in 2010 was the clinical tutor at the Graduate entry medical school in Royal College of Surgeons in Ireland. She has recently taken up a lecturer post in the Human Factors programme at the National Surgical Training Centre in Royal College of Surgeons in Ireland.

## Pre-publication history

The pre-publication history for this paper can be accessed here:

http://www.biomedcentral.com/1472-6920/13/38/prepub

## Supplementary Material

Additional file 1 An outline of the Personal and Professional Development course. Click here for file
