# Supplementary material for: Emotional intelligence assessment in a graduate entry medical school curriculum
Source: BMC Med Educ. 2013 Mar 7;13:38. doi: 10.1186/1472-6920-13-38 (PMC3599294; doi:10.1186/1472-6920-13-38)
Supplement: Additional file 1 — An outline of the Personal and Professional Development course. [file 1472-6920-13-38-S1.pdf]

Additional file 1: An outline of the Personal and Professional Development course

**Session title**	**Teaching methods**	**Content**	**Assessment methods**	**Teaching staff**
Professionalism 1	Small group work and presentation to class	Task for discussion in small groups: You have 30 minutes to consider the following questions. Present your answers as a PowerPoint presentation.<br>Q1.Decide on a definition of professionalism which you believe is relevant for the medical profession in the 21st century.<br>Q2.List some examples of medical student behaviour which does/does not demonstrate professionalism<br>Q3.What strategies do you think would help to facilitate medical students' sense of professionalism?	Eportfolio	Senior Lecturer/Clinical Psychologist, Clinical tutor/Physician, Clinical skills tutor
Professionalism 2	Didactic presentation	Demonstration of eportfolio and explanation of case uploads and reflective diary	Eportfolio. Clinical Competencies module.	Clinical skills tutor, Senior Lecturer/Clinical Psychologist
Stress 1	Small group discussion with online upload of group answers onto VLE (Moodle) and debrief in class.	Task for discussion in small groups: Consider the following questions and upload your answers onto your group forum on Moodle.<br>Q1. What are the signs of stress?<br>Q2. What are the sources of stress?		Senior Lecturer/Clinical Psychologist
Stress 2	Experiential	Large group practice of a Benson tension-reduction relaxation exercise on the floor/mat		Senior Lecturer/Clinical Psychologist
Stress 3	Practical demonstration and discussion	Cognitive strategies: The ABC model	<u>Assignment1:</u> Design a stress management programme for a patient (described) presenting to you with signs of stress.	Senior Lecturer/Clinical Psychologist
Leadership 1	Large group discussion and didactic presentation	Task for small group discussion: Consult relevant resources if desired and then upload your answers onto your group forum on Moodle.<br>Q1 What makes a good leader?		Senior Lecturer/Clinical Psychologist

		Q2 Leadership skills are an important aspect of a doctor's personal and professional development. **What, in your group's opinion, are the ten most crucial personal attributes/characteristics which determine the effectiveness of doctors as leaders?**		
Leadership 2	Large group discussion and didactic presentation	Leadership competencies. Individualised EI reports distributed and group feedback.		Senior Lecturer/Clinical Psychologist
Leadership 3	PowerPoint presentation and discussion.	Tips to develop emotional competencies	Assignment 2: Discuss your EI profile using a SWOT analysis (strengths/weaknesses/opportunities / threats)	Senior Lecturer/Clinical Psychologist
